# *meta*-Oligoazobiphenyls – synthesis via site-selective Mills reaction and photochemical properties

**DOI:** 10.3762/bjoc.8.99

**Published:** 2012-06-13

**Authors:** Raphael Reuter, Hermann A Wegner

**Affiliations:** 1 University of Basel, Department of Chemistry, St. Johanns-Ring 19, 4056 Basel, Switzerland

**Keywords:** azobenzene, cross-coupling reaction, foldamer, molecular switches, photochromism

## Abstract

The investigation of multi-photochromic compounds constitutes a great challenge, not only from a synthetic point of view, but also with respect to the analysis of the photochemical properties. In this context we designed a novel strategy to access *meta*-oligoazobiphenyls via site-selective Mills reaction and Suzuki cross-coupling in a highly efficient iterative way. Photochemical examination of the resulting monomeric and oligomeric azo compounds revealed that the overall degree of switching was independent of the connected azo-units. However, one of the azobonds in the bis-azobiphenyl is isomerized preferentially despite the high structural similarity.

## Introduction

The possibility to control structures on the molecular level in a reversible manner by using an external stimulus has fascinated scientists for a long time. One of the earliest reports on a molecular entity that can be influenced in this way concerns the azobenzene scaffold. Since this discovery by Hartley in 1937 [1] many more compounds showing such a photochromism have been reported [2]. However, the azobenzene moiety still remains one of the most popular “work horses” in this respect [3–6]. The reason can probably be found in its relatively easy synthetic accessibility combined with its interesting switching behavior. Upon irradiation with UV light the usually more stable *E* isomer can be switched to the *Z* form. If the *Z* isomer is irradiated with visible light or heated it can be converted back to the *E* state. During this isomerization the azobenzene undergoes a drastic length reduction of ~3.5 nm rendering it an ideal candidate for changing spatial arrangements on the molecular level. This change has been featured in a variety of applications [7–9], such as, switchable sensors [10], ion channels [11], catalysts [12], or liquid crystals [13].

**Figure 1:**
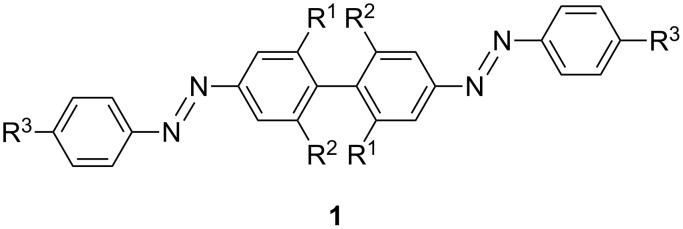
*para*-Substituted bisazobiphenyls **1** investigated by Hecht (R^1^, R^2^ = H, Me, R^3^ = *t*-Bu) and by Woolley and co-workers (R^1^ = H, SO_3_Na, R^2^ = H, R^3^ = NHCOCH_3_).

In most of these applications, however, only one azobenzene unit is incorporated. One of the reasons is the synthetic challenge associated with the preparation of such oligomers. Solubility and, especially, selectivity issues have to be addressed. Another reason is the higher complexity of the photochemistry of these multi-photochromic compounds. The number of possible isomers increases exponentially with the number of azo units. Additionally, it has been shown by others [14], as well as by us [15–17], that the direct connection of two azobonds to one aromatic ring alters the switching behavior considerably, such that bis-*ortho*-azobenzenes did not show any photochromicity. Therefore, in recent examples in the literature the azo-units have been dissected by incorporation of biphenyl units. Hecht and co-workers investigated the effect of the electronic coupling in detail and showed that the incorporation of *ortho*-methyl groups on the biphenyl restores the photoisomerization properties (Figure 1) [18–19]. In a related example by Samanta and Woolley a similar compound was presented featuring anchoring groups for biological applications (Figure 1) [20]. In another study the team of Hecht synthesized oligomers separated by alkynyl linkers, demonstrating interesting coil–uncoil phenomena upon irradiation [21]. There are also reports on polymers that contain multiple azobenzene units. In these materials, however, the effect of the individual azobenzene moiety on the whole molecular assembly is not specified [22–23].

So far, mainly *para*-connected bisazobiphenyls have been addressed. Synthetic studies on *meta*-bisazobiphenyls were reported early on, in the context of azodyes over 100 years ago [24–26]. The switching properties of this class of compounds have not been examined though. As it has been shown that the connectivity is crucial for the photochemical properties, we set out to study *meta*-connected azobiphenyls. Not only have the photochemical properties been investigated, but also selective synthetic strategies for their preparation have been explored.

## Results and Discussion

### Synthesis of *meta*-substituted azobiphenyls

**Figure 2:**
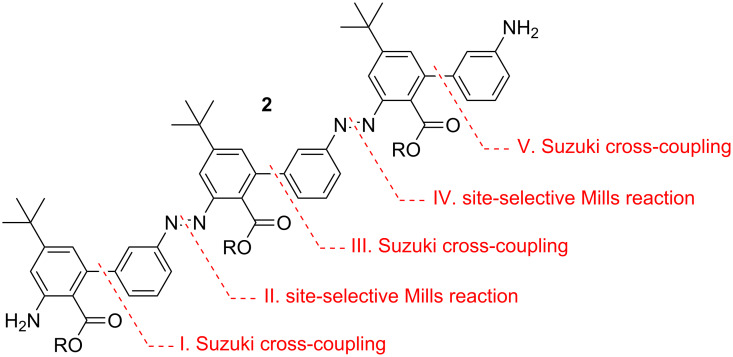
Synthetic strategy for the assembly of *meta*-substituted oligo-azobiphenyls **2**.

In the design of our target molecule, different aspects were considered: One important issue is solubility, which was addressed by incorporating *tert-*butyl-groups on every second ring. Additionally, it was envisioned to attach groups such as an amino functionality, which would open the potential to incorporate our system in, for example, biological settings. As a synthetic strategy it would be highly desirable to be able to build up the oligomer in a modular, protecting-group-free manner. In this respect, we planed a combination of the Mills reaction [27] with Suzuki cross-coupling, which we have successfully employed in previous studies (Figure 2) [28]. A key issue, however, was a site-selective transformation in order to differentiate both ends of the oligomer. The installment of an ester group on one site of the biphenyl unit should steer the critical Mills reactivity not only by steric, but also by electronic factors to the second ring.

**Scheme 1:**
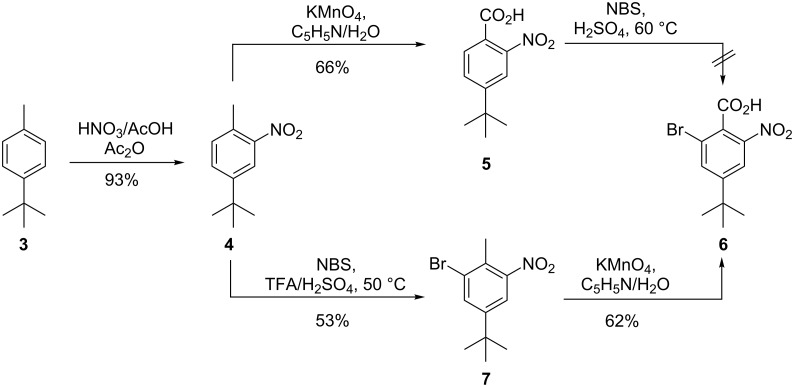
Synthesis of 2-nitro-4-*tert*-butyl-6-bromobenzoic acid (**6**).

One of the building blocks for the synthesis envisioned in Figure 2 is a tetra-substituted phenylderivative, which can be prepared from readily available 4-*tert*-butyltoluene (**3**) (Scheme 1). The first reaction was a nitration at the 2-position. After discouraging results were obtained under classical nitrating conditions employing a H_2_SO_4_/HNO_3_ mixture, the conditions were altered to use a mixture of AcOH/HNO_3_ in acetic anhydride as the solvent. In this way the mono nitration product **4** could be obtained in excellent yields. In a first attempt, the intermediate 2-nitro-4-*tert*-butyltoluene (**4**) was oxidized with potassium permanganate in a 1:1 solvent mixture of pyridine/H_2_O, to the corresponding benzoic acid **5**. The reaction proceeded with an acceptable yield of 66%. However, the resulting highly deactivated aromatic system **5** did not react even under harsh bromination conditions with NBS in concentrated sulfuric acid. Therefore, the reaction sequence was changed to bromination first, followed then by oxidation of the benzylic position. The bromination proceeded slowly, and after three days of stirring at 50 °C, only 53% of 2-nitro-4-*tert*-butyl-6-bromotoluene (**7**) was obtained. It turned out that the oxidation of this substrate was not as convenient as in the first attempt. Complete conversion of the starting material could not be achieved, even after stirring at 100 °C overnight and by using an excess of 10 equiv of KMnO_4_. Finally, the desired key compound **6** was isolated in a yield of 62%.

**Scheme 2:**
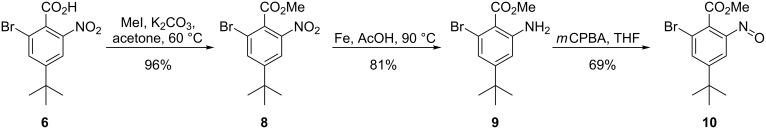
Preparation of nitroso derivative **10**.

The next two steps in the synthesis of the building blocks were straightforward (Scheme 2) and comprised esterification of the acid **6** with potassium carbonate and methyl iodide in acetone, as well as reduction of the nitro group via a Béchamp reaction to give compound **9**. The amino group was afterwards reacted with *m*CPBA in THF to yield the nitroso derivative **10** in a good yield of 69%.

**Scheme 3:**
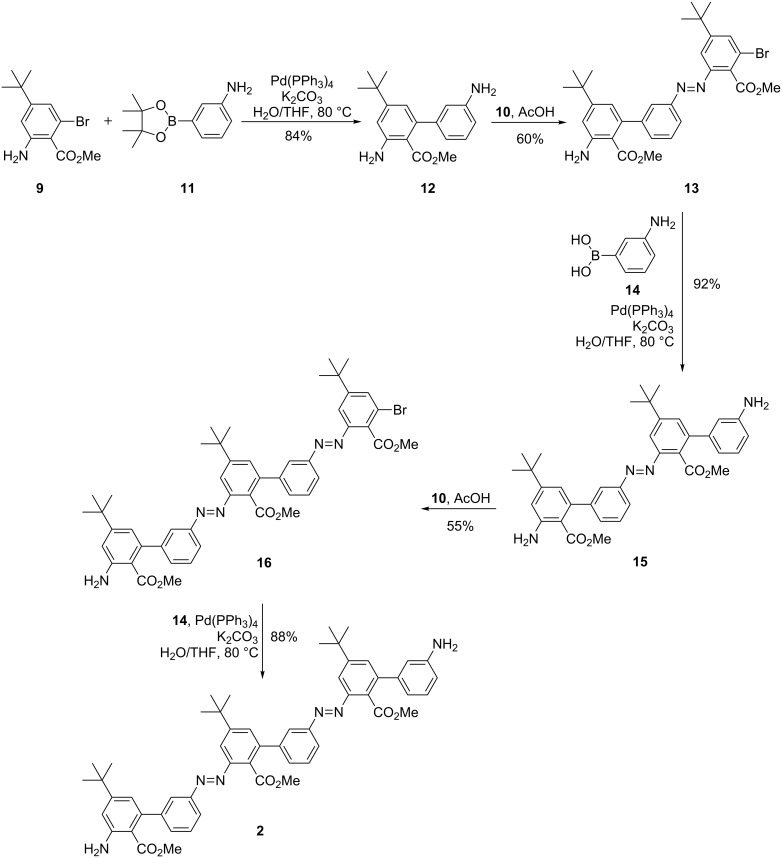
Assembly of oligomer **2** by Suzuki cross-coupling and site-selective Mills reaction.

With all the necessary building blocks in hand, the assembly of oligomer **2** was started with a Suzuki reaction of **9** with 3-aminophenylboronic acid pinacolate (**11**) to prepare biphenyl **12** (Scheme 3). The reaction proceeded in a good yield of 84%. However, pinacol was obtained as a side product and could not be separated by column chromatography. The impure diamine **12** was then subjected to the Mills reaction with one equiv of nitroso compound **10**. The aim was to achieve selectively only one coupling at the less functionalized benzene ring. As expected from the initial design, the deactivation of the second amino group by the ester in the *ortho*-position led to the isolation of the mono-coupled product as the major species in 60%. After this successful Mills reaction step, two more Suzuki reactions and one additional Mills coupling step were required to obtain the bisazobiphenyl **2**. The Suzuki reactions were performed with 3-aminophenylboronic acid (**14**) instead of the pinacolate, to eliminate the difficulties in the purification. The selective Mills reaction was again successful, giving only slightly lower yields.

### Isomerization studies

**Figure 3:**
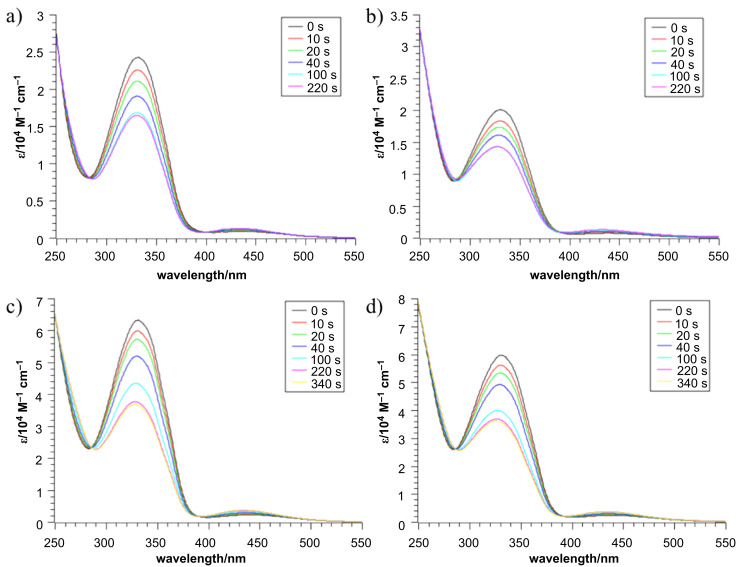
Isomerization studies of compound **13** (a), **15** (b), **16** (c) and **2** (d) (Irradiation at 356 nm in CHCl_3_).

Compounds **13**, **15**, **16** and **2** were analyzed by UV spectroscopy. All of them exhibit the typical behavior of azobenzenes, with a strong absorption at 330 nm for the π–π* transition and a weak absorption at 430 nm for the n–π* transition (Figure 3). For compounds **16** and **2** an increased absorption of the whole spectrum is noted due to the additional chromophore in the molecule. Upon irradiation at 356 nm (8 W hand-held UV lamp, at room temperature) all compounds underwent an *E*→*Z* isomerization, which can be seen as a decrease of the π–π* and an increase of the n–π* band. In this respect all compounds display similar characteristics and show a comparable degree of isomerization (Figure 3).

**Figure 4:**
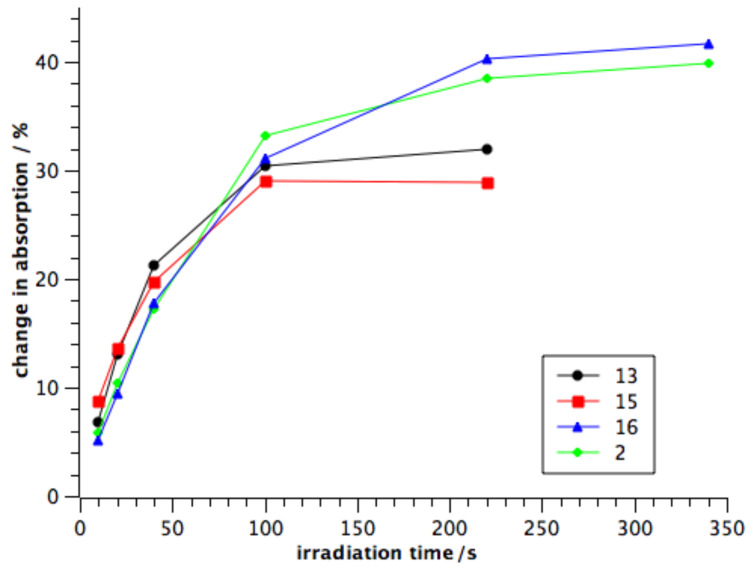
Comparison of the absorption as well as the photostationary state of compounds **13**, **15**, **16**, **2**.

This phenomenon can be easily visualized when the degree of isomerization is plotted against the irradiation time for all four compounds (Figure 4). In the first 100 s all four compounds isomerize at the same rate regardless how many azo units are present. After this time compounds **13** and **15** reach their photostationary state and their isomeric ratio does not change significantly. The bisazocompounds **16** and **2** with their additional azo moiety, however, show a further switching, which plateaus when both of their photochromic azo units have been equilibrated in the photostationary state.

**Figure 5:**
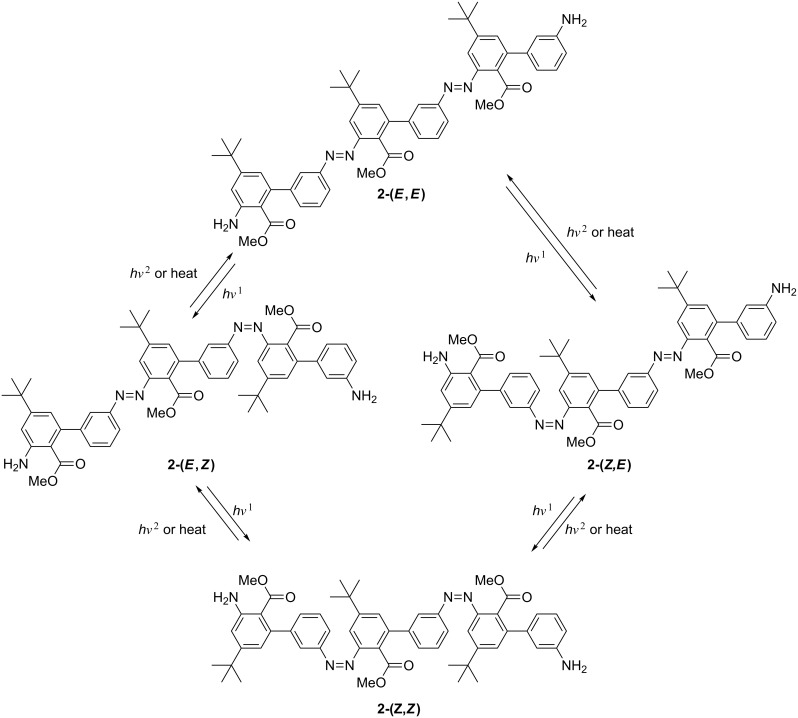
Four different isomers of **2**.

The composition of the photostationary state was investigated for compounds **15** and **2** by NMR spectroscopy. In the case of **15** the ratio of the *E*/*Z* isomers is 1/1.2 in the photostationary state corresponding to a degree of isomerization of ~55%. For **2** the situation is more complicated as, due to the lack of symmetry, four different isomers are possible [(*E*,*E*), (*E*,*Z*), (*Z*,*E*), (*Z*,*Z*); Figure 5]. The ratio in the photostationary state of (*E*,*E*)/(*E*,*Z*)/(*Z*,*E*)/(*Z*,*Z*) is 20/18/36/26, which corresponds also to a similar average isomerization of ~51% per azo unit. Although it may be expected that the (*E*,*Z*) and the (*Z*,*E*) isomer would be found in equal amounts as their direct chemical environment is very similar, a clear preference for the (*Z*,*E*) isomer can be seen. Therefore, although the overall degree of isomerization seems to be independent if two azo units are connected via a biphenyl unit in *meta*-positions, there seem to be subtle differences that influence the preference as to which azo bond is switched preferentially. Such a property will be highly useful in the design of selective, switchable, functional oligomers.

## Conclusion

In summary, a very efficient modular synthetic approach for the preparation of *meta*-oligoazobiphenyls has been developed relying on a site-selective Mills reaction and Suzuki cross-coupling. The switching behavior of the azo units seems to be rather independent from an electronic point of view. However, delicate aspects favor certain isomers, allowing the rational design of selective switchable functional oligomers in the future.

## Supporting Information

File 1 Experimental procedures and characterization data.
