# Supplementary material for: meta-Oligoazobiphenyls – synthesis via site-selective Mills reaction and photochemical properties
Source: Beilstein J Org Chem. 2012 Jun 13;8:877–83. doi: 10.3762/bjoc.8.99 (PMC3388877; doi:10.3762/bjoc.8.99)
Supplement: File 1 — Experimental procedures and characterization data. [file Beilstein_J_Org_Chem-08-877-s001.pdf]

Supporting Information

for

meta-Oligoazobiphenyls – synthesis via site-selective Mills reaction and photochemical properties

Raphael Reuter and Hermann A. Wegner*

Address: University of Basel, Department of Chemistry, St. Johanns-Ring 19, 4056 Basel, Switzerland

Email: Hermann A. Wegner* - hermann.wegner@unibas.ch

* Corresponding author

Experimental procedures and characterization data

Contents:

Experimental procedures	S2
NMR spectra	S9
Isomerization of **2**	S18
References	S19

Experimental procedures

2-Nitro-4-*tert*-butyltoluene (4)

Acetic acid (20.0 mL, 349 mmol, 4.32 equiv) was slowly dropped into ice-cooled fuming nitric acid (12.0 mL, 267 mmol, 3.30 equiv) with the temperature kept below 15 °C. The mixture was then added dropwise over 1 h to a solution of 4-*tert*-butyltoluene (13.8 mL, 80.0 mmol, 1.00 equiv) in acetic anhydride (400 mL). The mixture was stirred at rt for 2 h and poured onto ice water and extracted with DCM (2 × 100 mL). The extracts were slowly treated with 2 M sodium hydroxide, while being cooled in an ice bath. Then, the organic layer was separated, washed with water and brine (100 mL), dried over $MgSO_4$ and concentrated under reduced pressure to yield 14.5 g of an orange oil (93%).

^{1}H NMR: (400 MHz, $CDCl_3$, δ/ppm) 7.97 (s, 1H), 7.52 (d, $^3J_{HH}$ = 8.0 Hz, 1H), 7.26 (d, $^3J_{HH}$ = 8.0 Hz, 1H), 2.56 (s, 3H), 1.34 (s, 9H).

The analytical data correspond with the literature [1].

2-Nitro-4-*tert*-butylbenzoic acid (5)

A solution of 2-nitro-4-*tert*-butyltoluene (7.00 g, 36.2 mmol, 1.00 equiv) in pyridine (30 mL) and water (30 mL) was treated with potassium permanganate (28.6 g, 181 mmol, 5.00 equiv) and heated to 110 °C. After stirring for 2 h, another 5 equiv of potassium permanganate were added and the mixture was stirred at 110 °C for 16 h. Then, the mixture was filtered and the filter cake was washed with 2 M NaOH and ethyl acetate. The aqueous phase was collected and acidified with conc. aq. HCl and extracted with ethyl acetate. The combined organic phases were dried over $MgSO_4$ and the solvent was removed under reduced pressure to yield 5.34 g of a colorless solid (66%).

^{1}H NMR: (400 MHz, DMSO, δ/ppm) 7.91 (s, 1H), 7.83–7.79 (m, 2H), 1.32 (s, 9H).

The analytical data correspond with the literature [2].

2-Nitro-4-*tert*-butyl-6-bromotoluene (7)

A solution of 2-nitro-4-*tert*-butyltoluene (7.38 g, 38.2 mmol, 1.00 equiv) in TFA (23 mL) and conc. sulfuric acid (7.4 mL) was treated with NBS (13.6 g, 76.4 mmol, 2.00 equiv) in portions. After complete addition, the mixture was stirred at 50 °C for three days. Then, the mixture was poured onto ice water and extracted with ethyl acetate (3 × 50 mL). The combined organic extracts were washed with water and brine and dried over $MgSO_4$. After removal of the solvent, the residue was purified by column chromatography (silica gel, hexane/DCM 10:1) to obtain 5.54 g of a pale yellow oil (53%).

^{1}H NMR: (400 MHz, $CDCl_3$, δ/ppm) 7.78 (s, 1H), 7.71 (s, 1H), 2.52 (s, 3H), 1.33 (s, 9H).

The compound has been prepared before and characterized by elementary analysis [3].

2-Nitro-4-*tert*-butyl-6-bromobenzoic acid (6)

A solution of 2-nitro-4-*tert*-butyl-6-bromotoluene (3.35 g, 12.3 mmol, 1.00 equiv) in pyridine (10 mL) and water (10 mL) was treated with potassium permanganate (9.72 g, 61.5 mmol, 5.00 equiv) and heated to 110 °C. After stirring for 5 h another 5 equiv of potassium permanganate were added and the mixture was stirred at 110 °C for 15 h. Since the reaction was not complete, 2 equiv of $KMnO_4$ were added and stirring was continued for 2 h. Then, the mixture was filtered and the filter cake was washed with 2 M NaOH and ethyl acetate. The aqueous phase was collected and acidified with conc. aq. HCl and extracted with ethyl acetate. The combined organic phases were dried over $MgSO_4$ and the solvent was removed under reduced pressure to yield 2.34 g of a colorless solid (63%).

^{1}H NMR: (400 MHz, $CDCl_3$, δ/ppm) 8.20 (s, 1H), 7.93 (s, 1H), 1.38 (s, 9H).

^{13}C NMR: (101 MHz, $CDCl_3$, δ/ppm) 169.5, 155.9, 146.3, 135.8, 128.1, 120.7, 120.6, 35.5, 30.8.

Methyl 2-amino-4-*tert*-butyl-6-bromobenzoate (9)

To a solution of 2-nitro-4-*tert*-butyl-6-bromobenzoic acid (2.30 g, 7.61 mmol, 1.00 equiv) in acetone (30 mL), iodomethane (2.39 mL, 38.0 mmol, 5.00 equiv) and potassium carbonate (5.26 g, 38 mmol, 5.00 equiv) were added. The mixture was stirred for 2 h at 60 °C and then allowed to cool to room temperature. After filtration, the filtrate was diluted with EtOAc (50 mL) and washed with water (2 × 30 mL). The combined organic phases were dried over $MgSO_4$ and the solvent was removed under reduced pressure to yield 2.32 g of a pale yellow oil [96%, ^1H NMR: (400 MHz, $CDCl_3$, δ/ppm) 8.16 (s, 1H), 7.90 (s, 1H), 4.00 (s, 3H), 1.38 (s, 9H)]. The crude product was used in the next step without further purification. A solution of methyl 2-nitro-4-*tert*-butyl-6-bromobenzoate (2.23 g, 7.05 mmol, 1.00 equiv) in acetic acid (20 mL) was heated to 80 °C. Then, iron powder (3.13 g, 56 mmol, 7.95 equiv) was added slowly over 90 min to keep the temperature below 90 °C. After complete addition, the mixture was stirred for an additional 30 min, after which it was diluted with water (130 mL). After extraction with *tert*-BuOMe (2 × 100 mL), washing of the combined organic phases with water (70 mL), and drying over $MgSO_4$, the solvent was evaporated under reduced pressure, and the residue was purified by flash column chromatography (silica gel, hexane/EtOAc 5:1) to yield 1.64 g of the product as a yellow oil (81%).

^{1}H NMR: (400 MHz, $CDCl_3$, δ/ppm) 6.99 (s, 1H), 6.61 (s, 1H), 4.83 (br s, 2H), 3.91 (s, 3H), 1.25 (s, 9H).

^{13}C NMR: (101 MHz, $CDCl_3$, δ/ppm) 167.9, 156.2, 148.7, 122.0, 121.1, 113.9, 112.7, 51.8, 34.8, 30.8.

MS: (FAB) *m*/*z* (%): 326 (17) $[M + 2 + K]^+$, 324 (16) $[M + K]^+$, 287 (89) $[M + 2]^+$, 285 (100) $[M]^+$.

Methyl 2-nitroso-4-*tert*-butyl-6-bromobenzoate (10)

A solution of methyl 2-amino-4-*tert*-butyl-6-bromobenzoate (1.08 g, 3.78 mmol, 1.00 equiv) and *m*CPBA (1.30 g, 7.56 mmol, 2.00 equiv) was stirred at rt for 3 h. Then, the solvent was removed at reduced pressure and the residue was purified by column chromatography (silica gel, hexane/DCM 10:1 to hexane/DCM 4:1) to yield 780 mg of a green oil (69%).

^{1}H NMR: (400 MHz, $CDCl_3$, δ/ppm) 7.93 (s, 1H), 7.62 (s, 1H), 4.08 (s, 3H), 1.37 (s, 9H).

^{13}C NMR: (101 MHz, $CDCl_3$, δ/ppm) 166.8, 161.9, 155.7, 136.1, 128.5, 121.2, 115.2, 53.3, 35.4, 30.9.

Diaminobiphenyl 12

Methyl 2-amino-4-*tert*-butyl-6-bromobenzoate (501 mg, 1.75 mmol, 1.00 equiv) and 3-aminophenylboronic acid pinacolate (575 mg, 2.63 mmol, 1.50 equiv) were dissolved in THF (80 mL), and 2 M aq. K_2CO_3 was added (20 mL). The mixture was degassed by an argon stream for 15 min, and $Pd(PPh_3)_4$ (61 mg, 53 μmol, 3.0 mol %) was added. The mixture was stirred at 80 °C under an argon atmosphere for 20 h and then allowed to cool to rt. The organic phase was separated and washed with brine (40 mL). After drying over $MgSO_4$ and removal of the solvent under reduced pressure, the residue was purified by flash column chromatography (silica gel, hexane/EtOAc 3:1 to 2:1) to yield 560 mg of a colorless oil, which still contained pinacol (84%, purity: 78%).

^{1}H NMR: (400 MHz, $CDCl_3$, δ/ppm) 7.13 (t, $^3J_{HH}$ = 7.6 Hz, 1H), 6.71 (d, $^4J_{HH}$ = 1.8 Hz, 1H), 6.69–6.61 (m, 4H), 4.93 (br s, 2H), 3.69 (br s, 2H), 3.45 (s, 3H), 1.29 (s, 9H).

^{13}C NMR: (101 MHz, $CDCl_3$, δ/ppm) 170.1, 155.1, 147.8, 146.1, 144.6, 143.7, 128.8, 118.7, 117.7, 114.7, 113.6, 112.4, 111.7, 51.3, 34.8, 31.0.

Monoazobromoamine 13

A solution of diaminobiphenyl **12** (540 mg, 1.41 mmol, 1.00 equiv), and methyl 2-nitroso-4-*tert*-butyl-6-bromobenzoate (423 mg, 1.41 mmol, 1.00 equiv) in glacial acetic acid (50 mL) was stirred for 3 d at rt. The solvent was removed under reduced pressure and the residue was subjected to flash column chromatography (silica gel, hexane/TBME 2:1) to yield 490 mg of an orange solid (60%).

^{1}H NMR: (400 MHz, $CDCl_3$, δ/ppm) 7.88 (d, $^4J_{HH}$ = 1.7 Hz, 1H), 7.84–7.79 (m, 2H), 7.69 (d, $^4J_{HH}$ = 1.7 Hz, 1H), 7.50 (t, $^3J_{HH}$ = 7.7 Hz, 1H), 7.42 (d, $^3J_{HH}$ = 7.7 Hz, 1H), 6.74–6.72 (m, 2H), 5.17 (br s, 2H), 3.98 (s, 3H), 3.40 (s, 3H), 1.37 (s, 9H), 1.31 (s, 9H).

^{13}C NMR: (101 MHz, $CDCl_3$, δ/ppm): 169.5, 167.6, 155.6, 155.0, 151.8, 150.1, 148.7, 144.8, 142.9, 131.8, 131.5, 131.0, 128.6, 122.9, 121.3, 119.9, 117.9, 116.0, 113.0, 110.7, 52.7, 51.2, 35.3, 34.9, 31.1, 31.0.

MS: (FAB) *m*/*z* (%): 620 (43) $[M]^+$, 581 (100) $[M]^+$.

Monoazodiamine 15

Monoazobromoamine **13** (450 mg, 775 µmol, 1.00 equiv) and 3-aminophenylboronic acid monohydrate (132 mg, 853 µmol, 1.10 equiv) were dissolved in THF (32 mL), and 2 M aq. K_2CO_3 was added (8 mL). The mixture was degassed by an argon stream for 15 min, and $Pd(PPh_3)_4$ (27 mg, 23 µmol, 3.0 mol %) was added. The mixture was stirred at 80 °C under an argon atmosphere for 19 h and then allowed to cool to rt. The organic phase was separated and washed with brine (16 mL). After drying over $MgSO_4$ and removal of the solvent under reduced pressure, the residue was purified by flash column chromatography (silica gel, hexane/TBME 1:3) to yield 423 mg of an orange solid (92%).

^{1}H NMR: (400 MHz, $CDCl_3$, δ/ppm) 7.87 (d, $^4J_{HH}$ = 1.8 Hz, 1H), 7.86–7.81 (m, 2H), 7.53–7.46 (m, 2H), 7.40 (d, $^3J_{HH}$ = 7.7 Hz, 1H), 7.21 (t, $^3J_{HH}$ = 7.8 Hz, 1H), 6.87 (d, $^3J_{HH}$ = 7.9 Hz, 1H), 6.80 (s, 1H), 6.76–6.68 (m, 3H), 5.14 (br s, 2H), 3.75 (br s, 2H), 3.74 (s, 3H), 3.41 (s, 3H), 1.40 (s, 9H), 1.31 (s, 9H).

^{13}C NMR: (101 MHz, $CDCl_3$, δ/ppm) 169.6, 169.2, 155.5, 153.4, 152.2, 149.3, 148.7, 146.5, 144.7, 143.0, 141.1, 140.6, 131.0, 129.8, 129.41, 129.36, 128.5, 122.9, 121.2, 118.9, 118.0, 115.2, 114.6, 113.7, 112.9, 110.8, 52.2, 51.2, 35.3, 34.9, 31.2, 31.0.

MS: (FAB) *m*/*z* (%): 594 (37) $[M]^+$, 592 (100).

Bisazobromoamine 16

A solution of monoazodiamine **15** (412 mg, 695 µmol, 1.00 equiv), and methyl 2-nitroso-4-*tert*-butyl-6-bromobenzoate (219 mg, 730 µmol, 1.05 equiv) in glacial acetic acid (20 mL) was stirred for 16 h at rt. The solvent was removed under reduced pressure and the residue was subjected to flash column chromatography (silica gel, hexane/TBME 2:1) to yield 333 mg of an orange solid (55%).

^{1}H NMR: (400 MHz, $CDCl_3$, δ/ppm) 8.01 (s, 1H), 7.94 (d, $^4J_{HH}$ = 1.8 Hz, 1H), 7.93–7.83 (m, 4H), 7.70 (d, $^4J_{HH}$ = 1.7 Hz, 1H), 7.66–7.56 (m, 3H), 7.53–7.48 (m, 1H), 7.41 (d, $^3J_{HH}$ = 7.5 Hz, 1H), 6.74 (dd, $^3J_{HH}$ = 7.7 Hz, $^4J_{HH}$ = 1.8 Hz, 2H), 5.15 (br s, 2H), 3.98 (s, 3H), 3.74 (s, 3H), 3.42 (s, 3H), 1.43 (s, 9H), 1.38 (s, 9H), 1.31 (s, 9H).

^{13}C NMR: (101 MHz, $CDCl_3$, δ/ppm) 169.6, 169.0, 167.5, 155.5, 155.1, 153.7, 152.2, 152.0, 150.1, 149.5, 148.7, 144.7, 143.0, 141.1, 139.4, 132.0, 131.9, 131.1, 130.8, 129.8, 129.35, 129.30, 128.5, 123.5, 122.9, 122.4, 121.3, 120.0, 117.9, 116.3, 114.4, 112.9, 110.8, 52.7, 52.2, 51.2, 35.3 (2C), 34.9, 31.2, 31.04, 30.95.

MS: (FAB) *m*/*z* (%): 914 (39) $[M + 2 + K]^+$, 912 (31) $[M + K]^+$, 57 (100).

Bisazodiamine 2

Bisazobromoamine **16** (320 mg, 366 µmol, 1.00 equiv) and 3-aminophenylboronic acid monohydrate (68.1 mg, 439 µmol, 1.20 equiv) were dissolved in THF (16 mL) and 2 M aq. K_2CO_3 was added (4 mL). The mixture was degassed by an argon stream for 15 min and $Pd(PPh_3)_4$ (13 mg, 11 µmol, 3.0 mol %) was added. The mixture was stirred at 80 °C under an argon atmosphere for 19 h and then allowed to cool. The organic phase was separated and washed with brine (8 mL). After drying over $MgSO_4$ and removal of the solvent under reduced pressure, the residue was purified by flash column chromatography (silica gel, hexane/TBME 1:4) to yield 287 mg of an orange solid (88%).

^{1}H NMR: (400 MHz, $CDCl_3$, δ/ppm) 8.03 (s, 1H), 7.94–7.90 (m, 2H), 7.88–7.82 (m, 3H), 7.63–7.47 (m, 5H), 7.40 (d, $^3J_{HH}$ = 7.5 Hz, 1H), 7.20 (t, $^3J_{HH}$ = 7.8 Hz, 1H), 6.87 (d, $^3J_{HH}$ = 7.6 Hz, 1H), 6.80 (s, 1H), 6.75–6.68 (m, 3H), 5.15 (br s, 2H), 3.75 (br s, 2H), 3.74 (s, 3H), 3.73 (s, 3H), 3.41 (s, 3H), 1.43 (s, 9H), 1.40 (s, 9H), 1.31 (s, 9H).

^{13}C NMR: (101 MHz, $CDCl_3$, δ/ppm) 169.9, 169.2, 169.0, 155.5, 153.7, 153.4, 152.5, 152.2, 149.5, 149.3, 148.7, 146.5, 144.7, 143.0, 141.1, 141.0, 140.6, 139.6, 131.4, 131.0, 129.9, 129.7, 129.6, 129.44, 129.36, 129.2, 128.5, 123.5, 122.9, 122.4, 121.3, 118.9, 117.9, 115.2, 114.6, 114.3, 113.9, 112.9, 110.8, 52.23, 52.15, 51.2, 35.31, 35.25, 34.9, 31.25, 31.23, 31.0.

MS: (FAB) *m*/*z* (%): 886 (48) $[M]^+$, 57 (100).

NMR-spectra:

2-Nitro-4-*tert*-butyl-6-bromobenzoic acid (6)

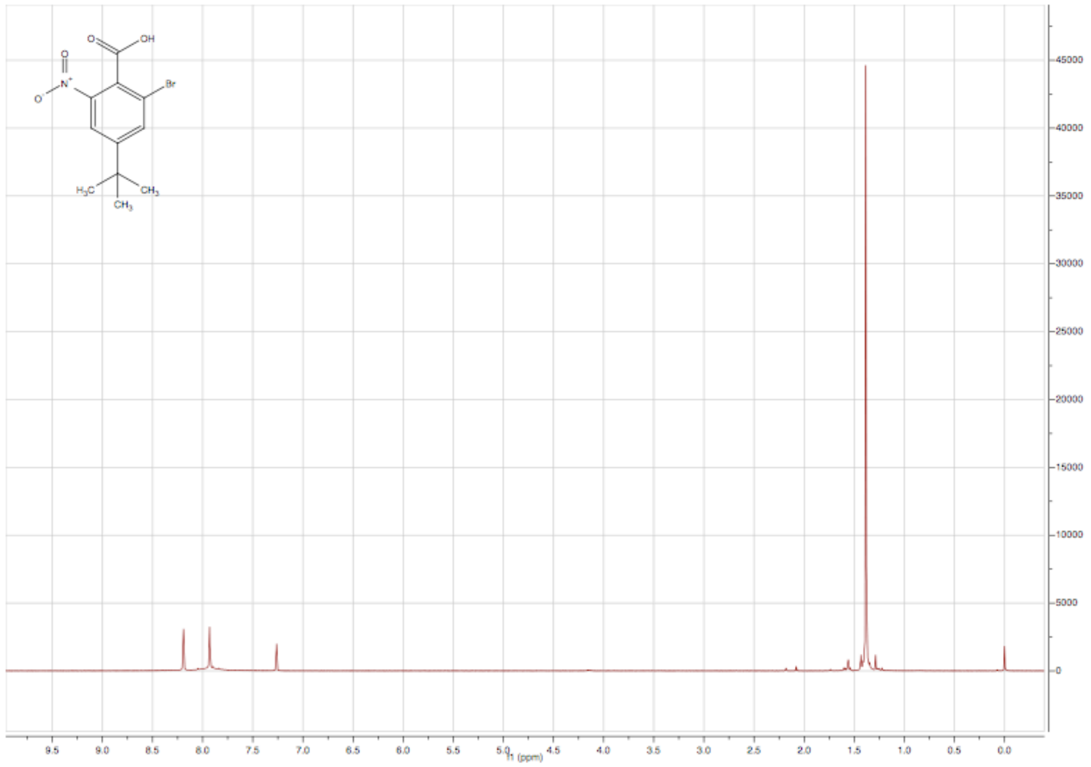

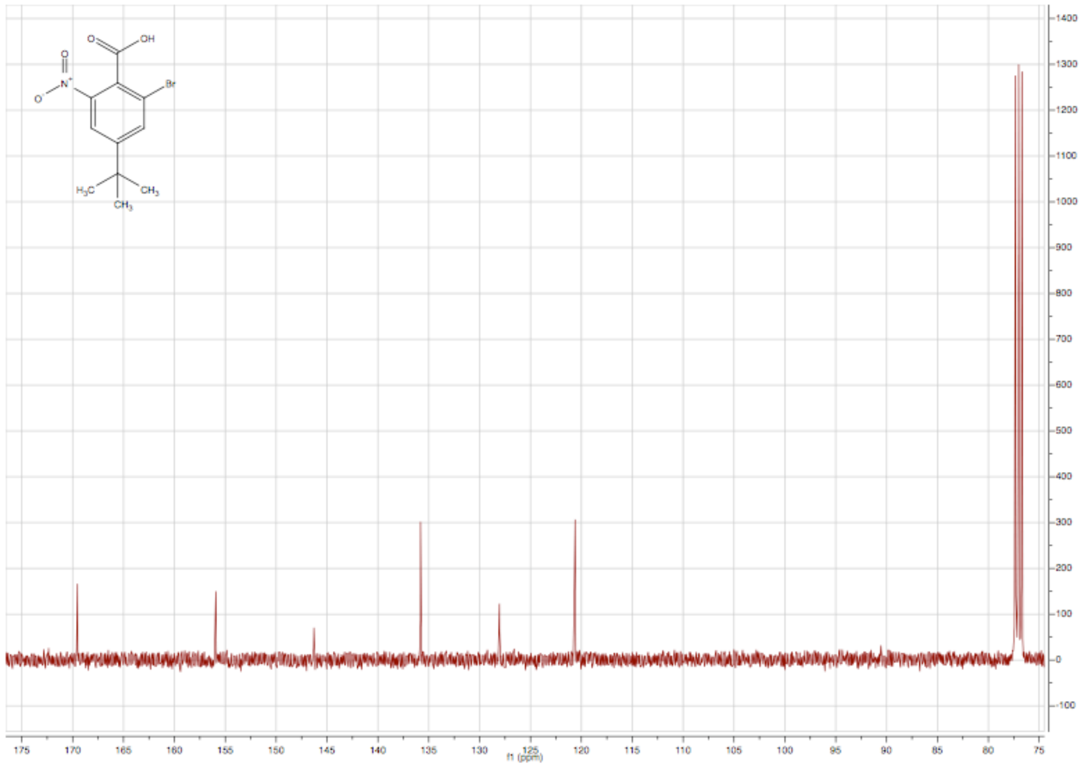

Methyl 2-nitro-4-*tert*-butyl-6-bromobenzoate (8)

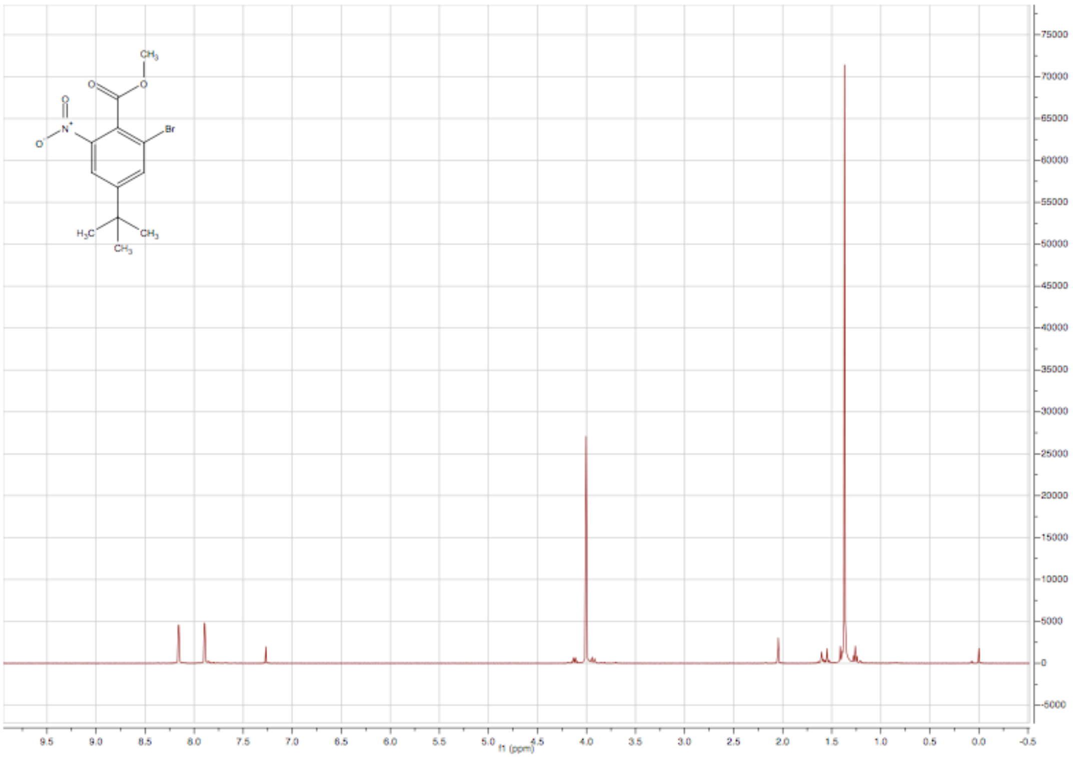

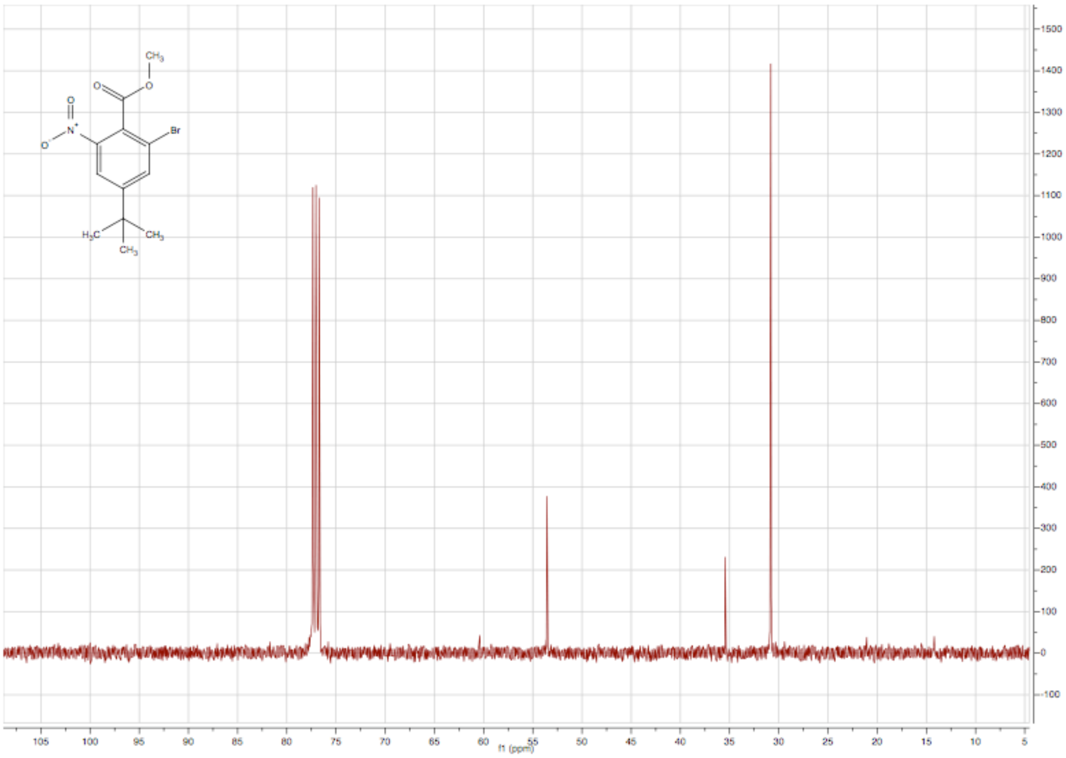

Methyl 2-amino-4-*tert*-butyl-6-bromobenzoate (9)

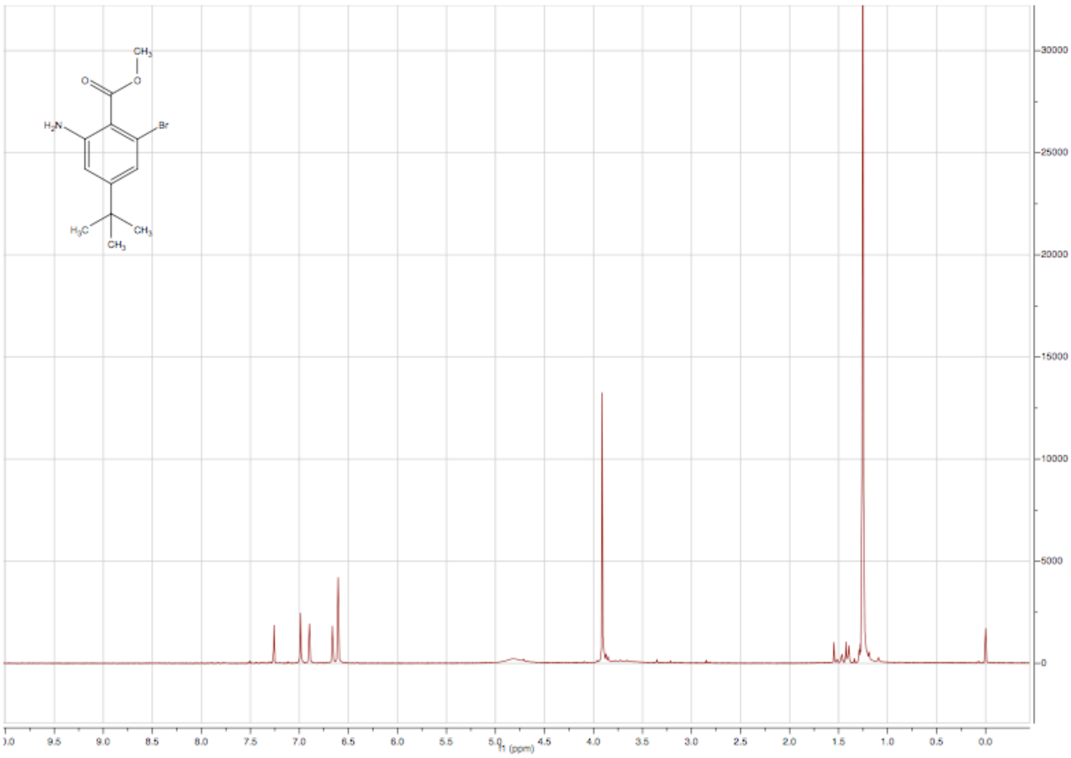

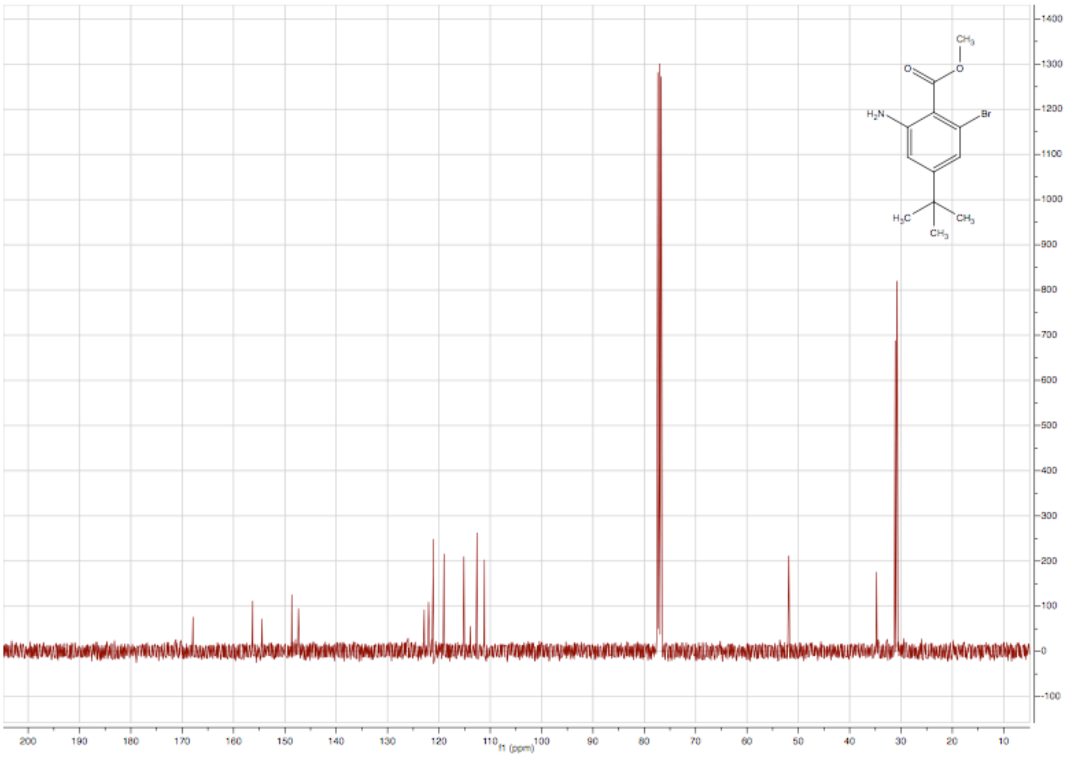

Methyl 2-nitroso-4-*tert*-butyl-6-bromobenzoate (10)

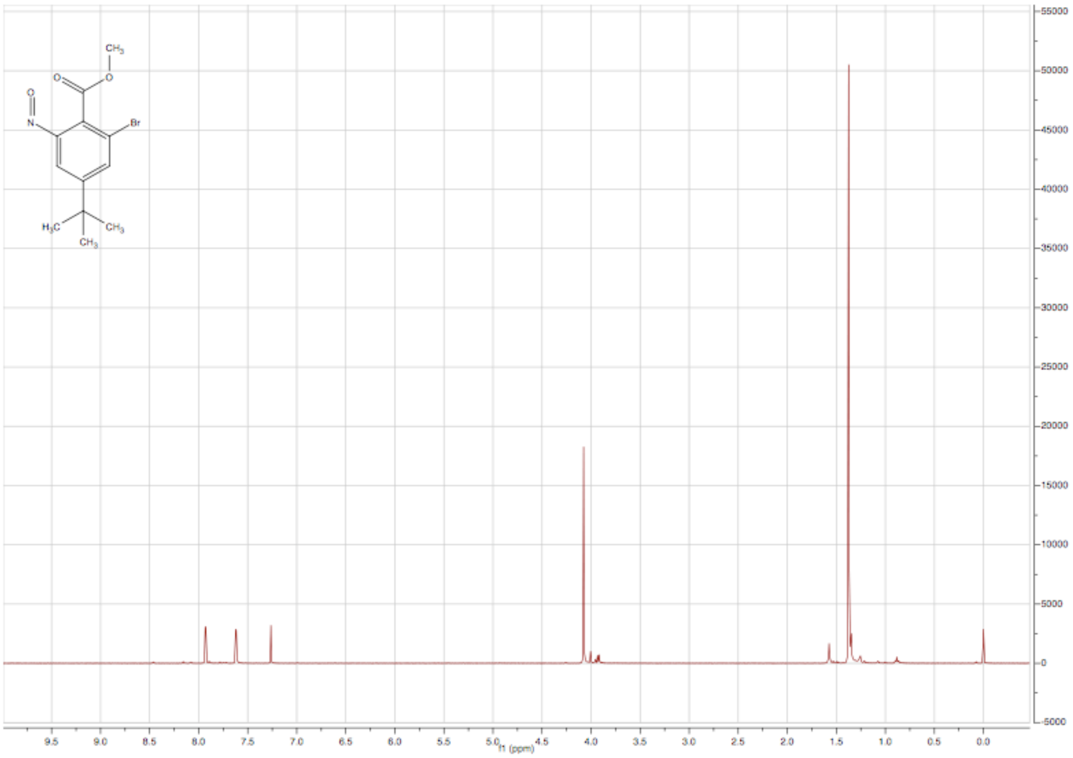

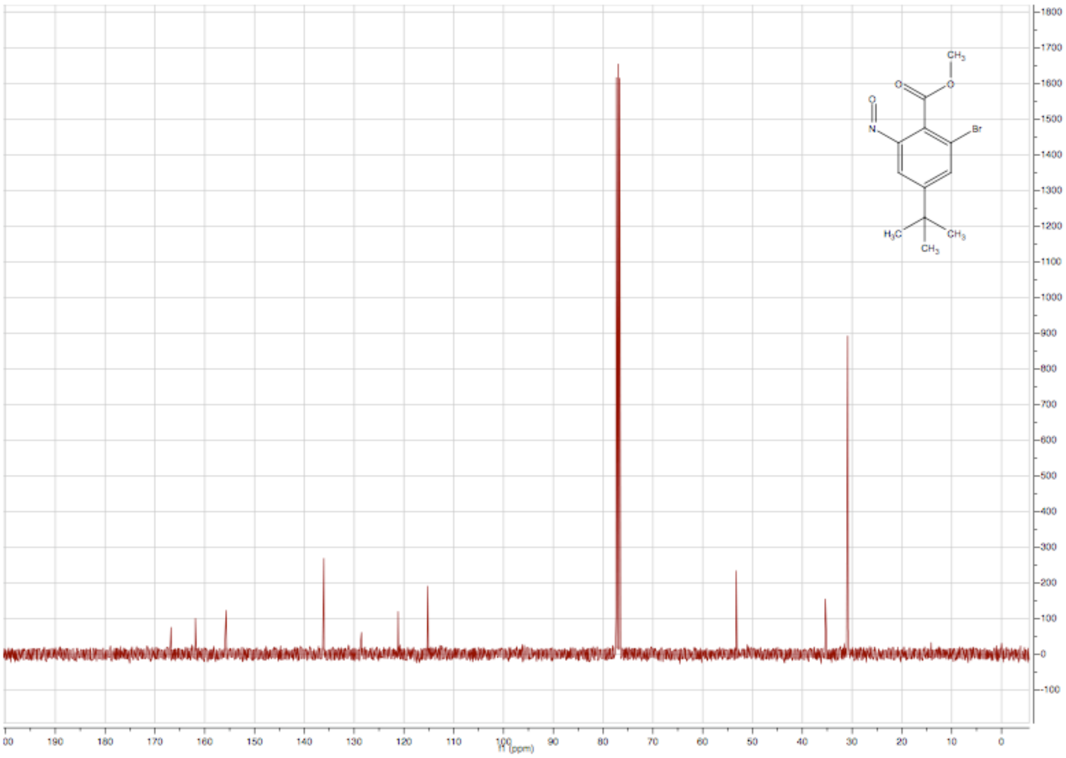

Diaminobiphenyl 12

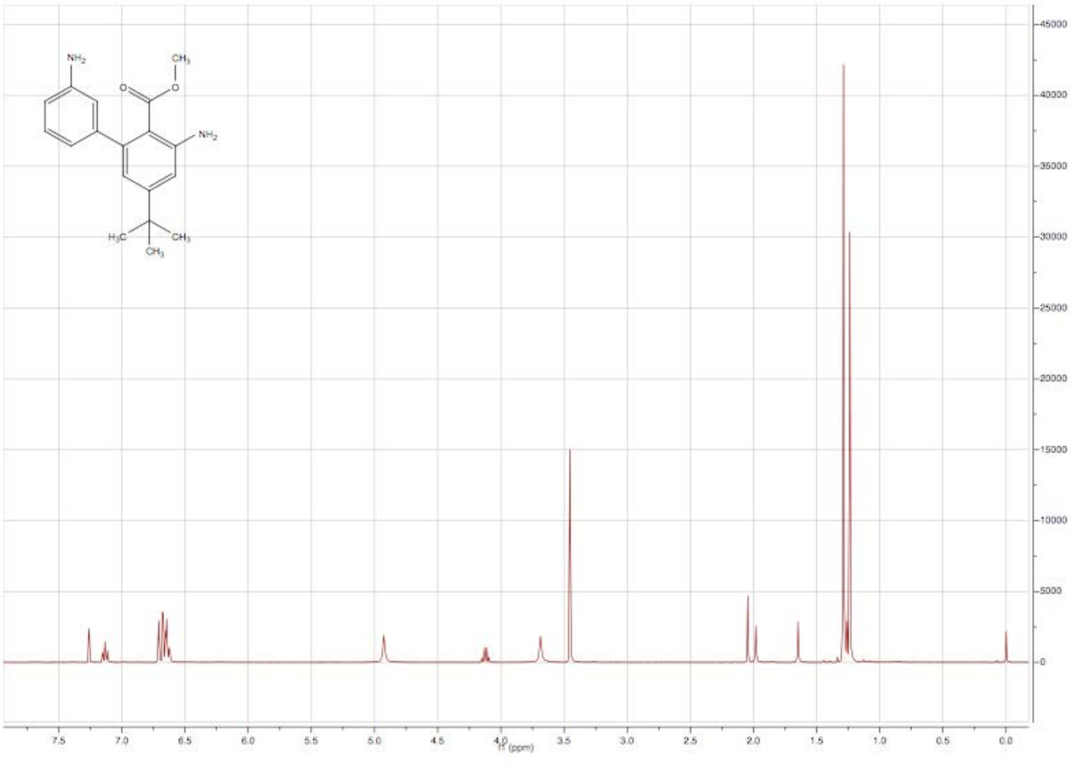

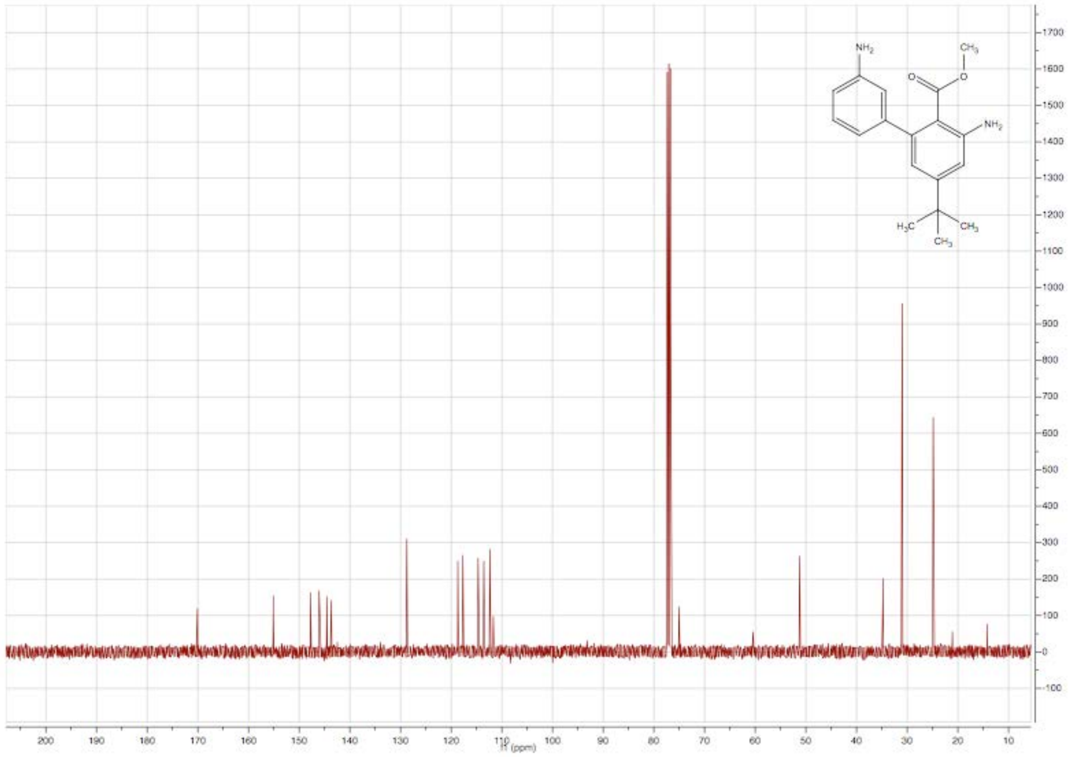

Monoazobromoamine 13

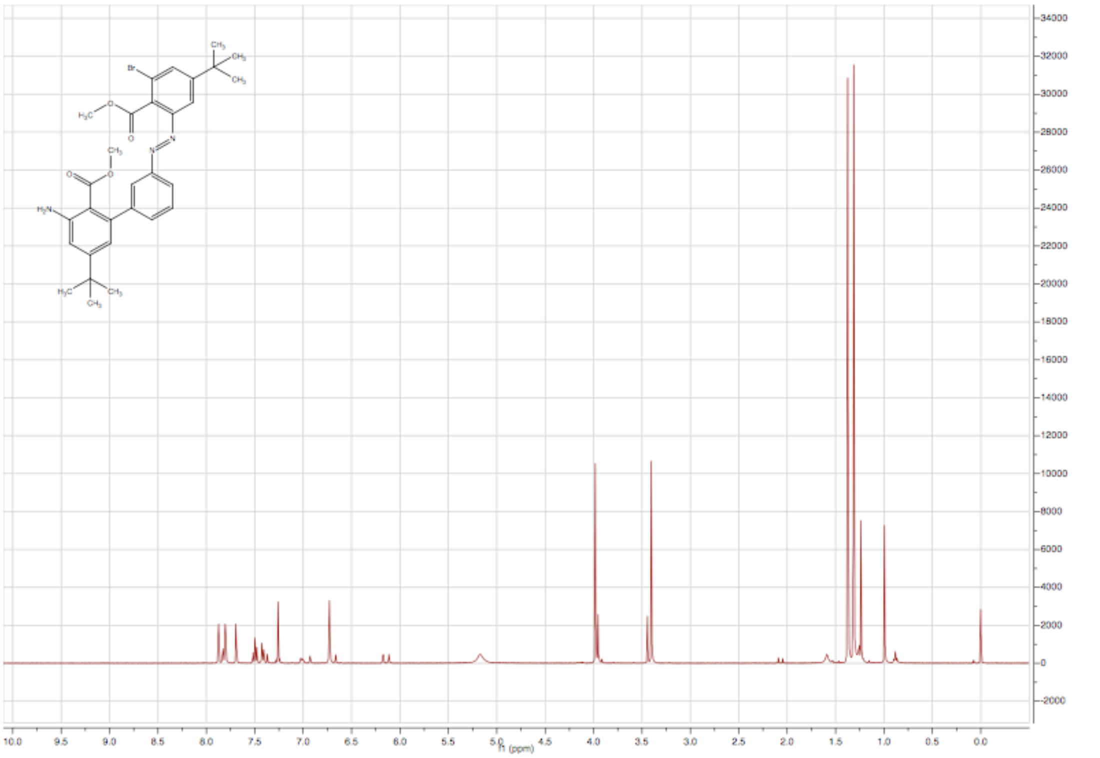

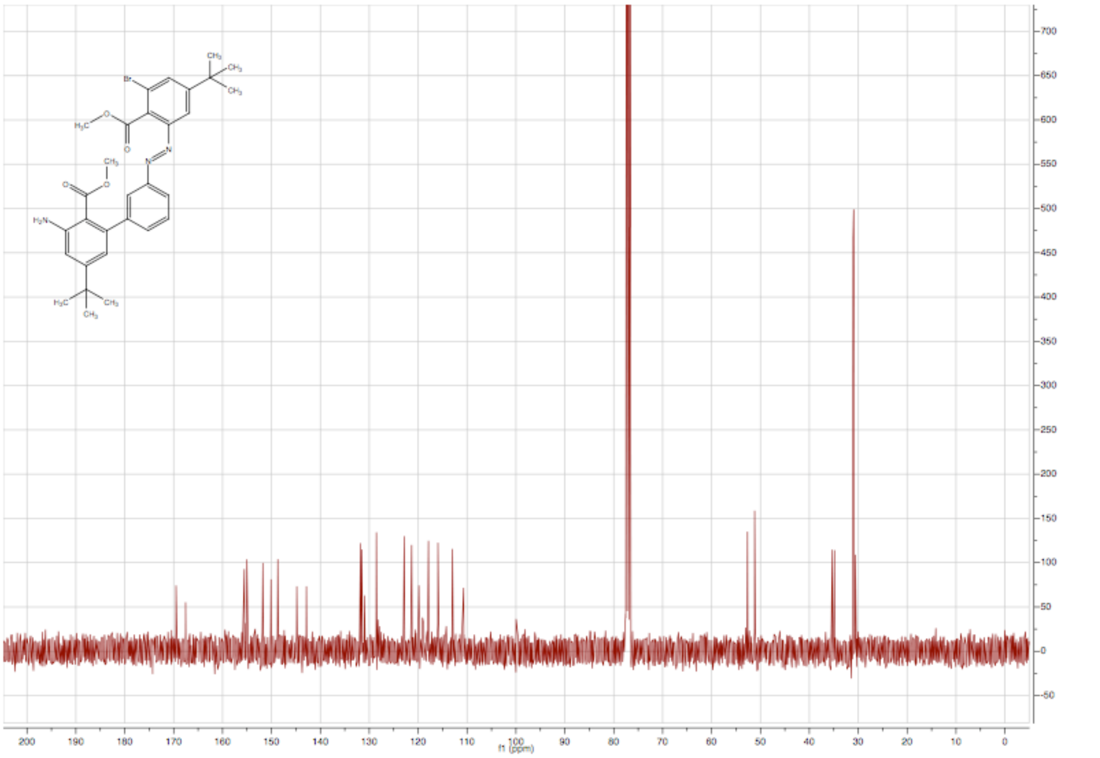

Monoazodiamine 15

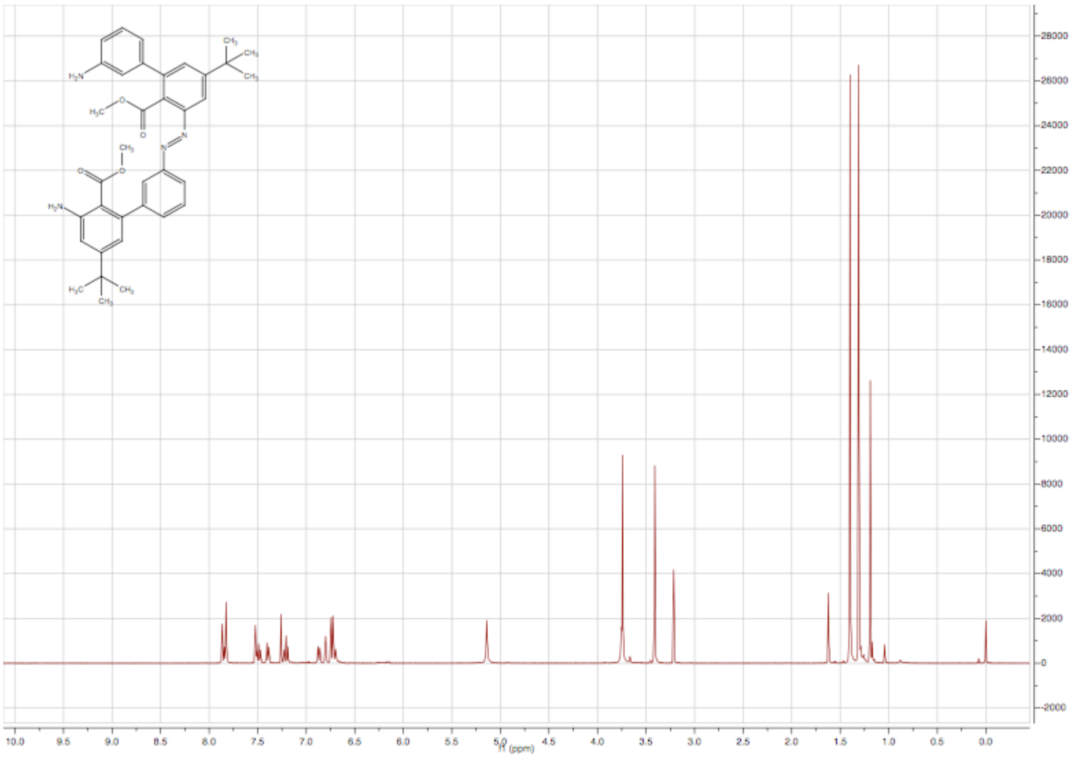

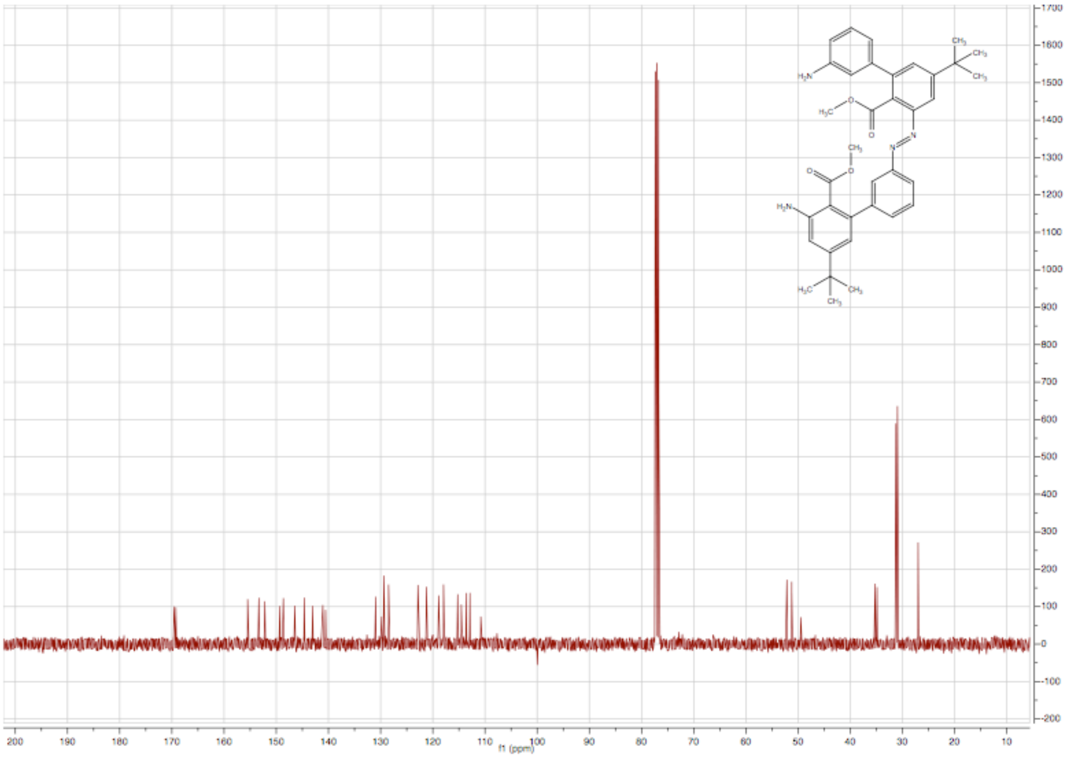

Bisazobromoamine 16

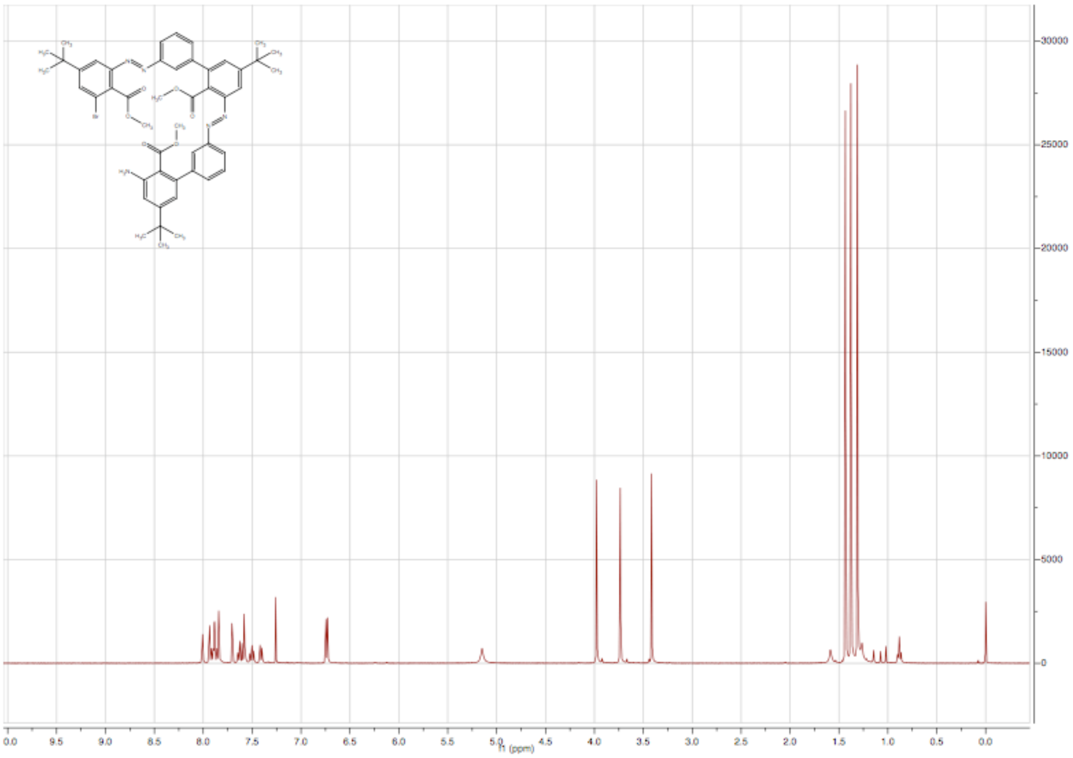

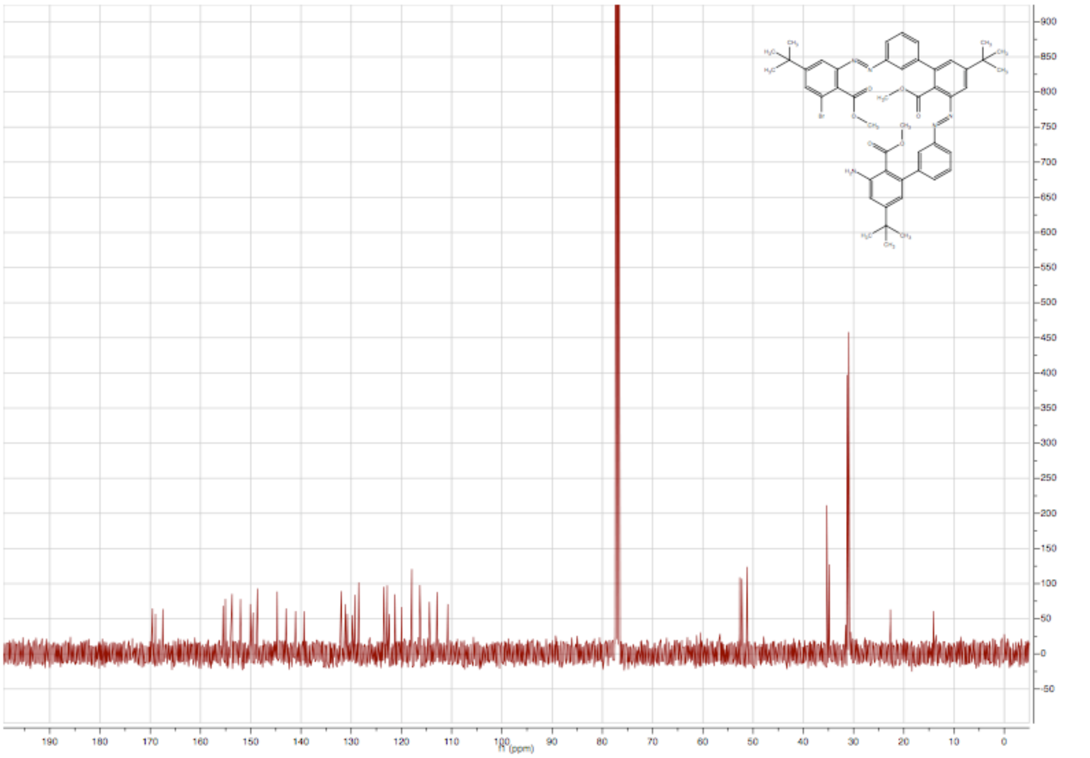

Bisazodiamine 2

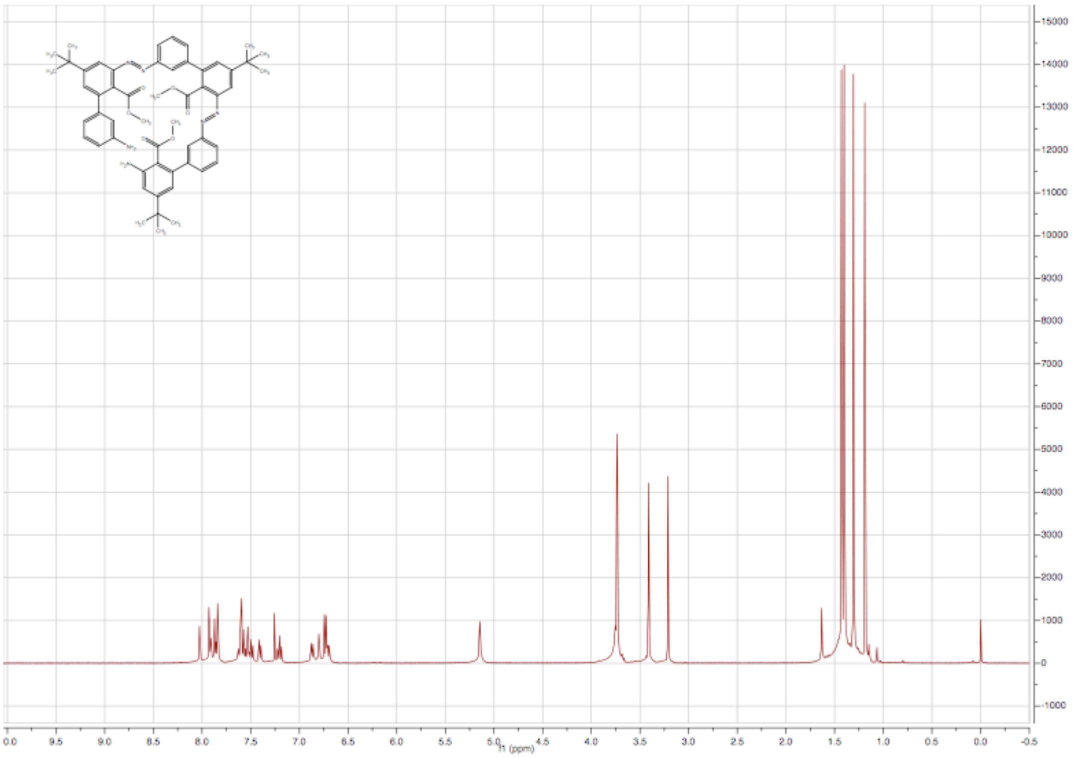

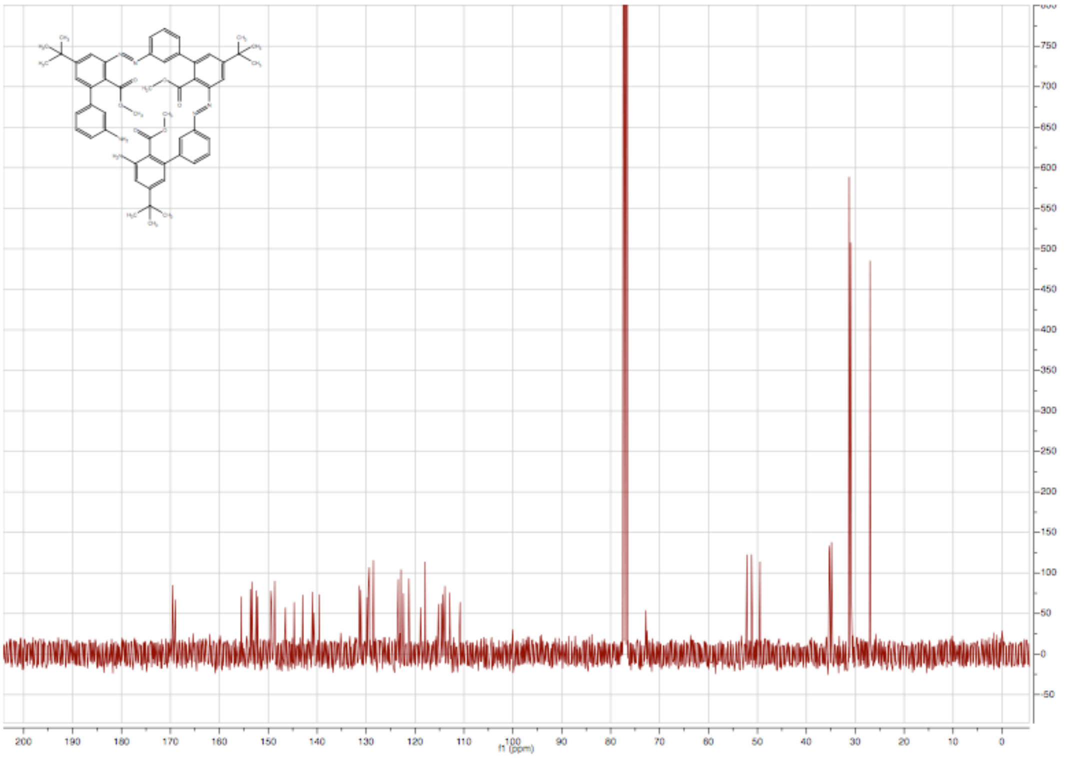

Isomerization of **2** (356 nm, rt):

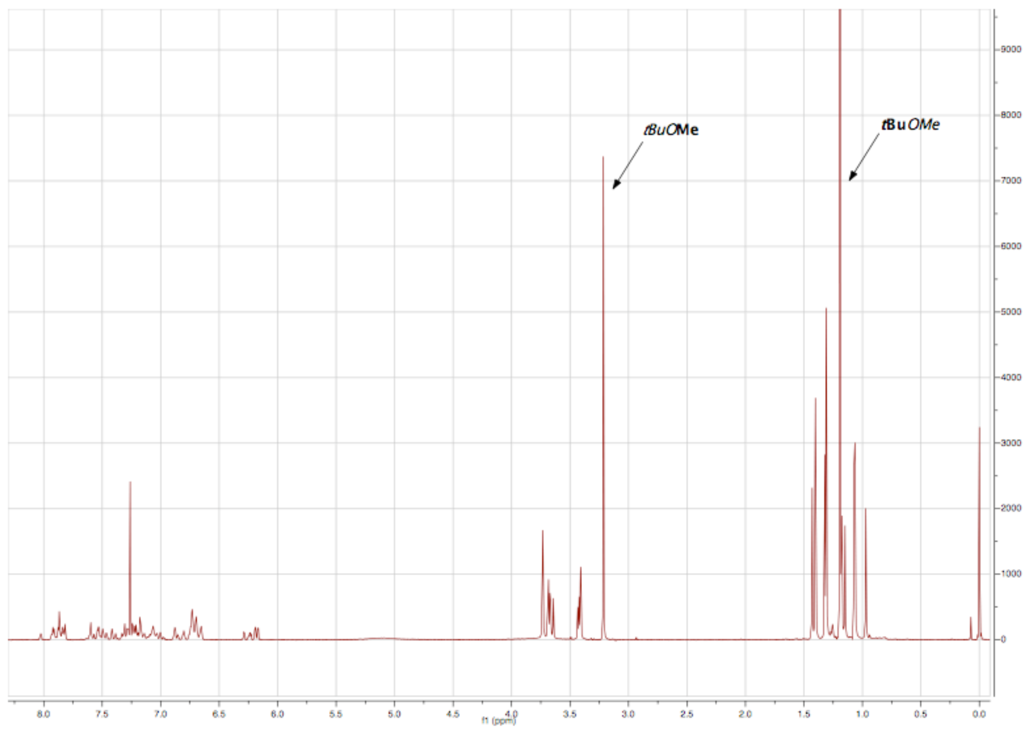

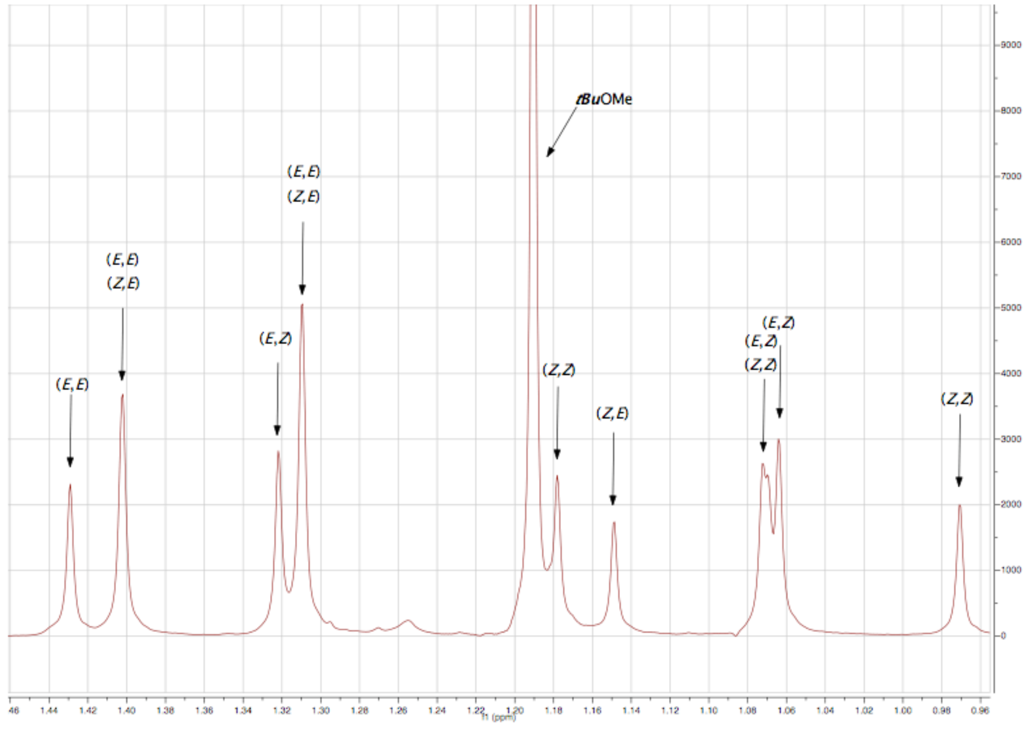

References

[1] Quideau, S.; Pouysegu, L.; Ozanne, A.; Gagnepain, J. *Molecules* **2005**, 10, 201-216.

[2] Brydon, D. L.; Cadogan, J. I. G.; Cook, J.; Harger, M. J. P.; Sharp J. T. *J. Chem. Soc. B* **1971**, 1996-2006.

[3] Tashiro, M.; Tsuzuki, H.; Matsumoto, J.-i.; Mataka, S.; Nakayama, K.; Tsurata, Y.; Yonemitsu, T. *J. Chem. Research (M)* **1989**, 2826-2851.
